# Impact of liberal *versus* conservative saturation targets on gas exchange indices in COVID-19 related acute respiratory distress syndrome: a physiological study

**DOI:** 10.5935/0103-507X.20210081

**Published:** 2021

**Authors:** Javier Hernán Dorado, Joaquín Pérez, Emiliano Navarro, Emiliano Gogniat, Sebastían Torres, Sabrina Cagide, Matías Accoce

**Affiliations:** 1 Sanatorio Anchorena San Martín - Buenos Aires, Argentina.; 2 Centro del Parque - Buenos Aires, Argentina.; 3 Capítulo de Kinesiología Intensivista, Sociedad Argentina de Terapia Intensiva -Buenos Aires, Argentina.; 4 Facultad de Medicina y Ciencias de la Salud, Universidad Abierta Interamericana - Buenos Aires, Argentina.

**Keywords:** COVID-19, Respiratory distress syndrome, Respiration, artificial, Oxygenation, Pulmonary gas exchange, Respiratory mechanics

## Abstract

**Objective:**

To compare gas exchange indices behavior by using liberal *versus* conservative oxygenation targets in patients with moderate to severe acute respiratory distress syndrome secondary to COVID-19 under invasive mechanical ventilation. We also assessed the influence of high FiO_2_ on respiratory system mechanics.

**Methods:**

We prospectively included consecutive patients aged over 18 years old with a diagnosis of COVID-19 and moderate-severe acute respiratory distress syndrome. For each patient, we randomly applied two FiO_2_ protocols to achieve SpO_2_ 88% - 92% or 96%. We assessed oxygenation indices and respiratory system mechanics.

**Results:**

We enrolled 15 patients. All the oxygenation indices were significantly affected by the FiO_2_ strategy (p < 0.05) selected. The PaO_2_/FiO_2_ deteriorated, PA-aO_2_ increased and Pa/AO_2_ decreased significantly when using FiO_2_ to achieve SpO_2_ 96%. Conversely, the functional shunt fraction was reduced. Respiratory mechanics were not affected by the FiO_2_ strategy.

**Conclusion:**

A strategy aimed at liberal oxygenation targets significantly deteriorated gas exchange indices, except for functional shunt, in COVID-19-related acute respiratory distress syndrome. The respiratory system mechanics were not altered by the FiO_2_ strategy.

**Clinical Trials Register:** NCT04486729.

## INTRODUCTION

The novel infection caused by coronavirus-19 (COVID-19) has been recently recognized and has spread throughout China and most countries around the world. Almost 25 million people were infected worldwide by August 27, 2020, and the number of deaths has risen to more than 820,000.^(^1^)^

Approximately 85% of COVID-19-infected patients admitted to the intensive care unit (ICU) develop severe acute respiratory syndrome (SARS-CoV-2).^(^2^)^ However, despite meeting acute respiratory distress syndrome (ARDS) criteria, the pathophysiological features and clinical course of SARS-CoV-2 may differ substantially from those of classical ARDS.^(^3^,^4^)^

According to the Berlin definition, the severity of ARDS is determined by the degree of gas exchange compromise.^(^3^)^ Consequently, the quantification of oxygenation indices is considered mandatory. In particular, the quotient of partial pressure of oxygen and fraction of inspired oxygen (PaO_2_/FiO_2_) is the oxygenation index most widely used in daily clinical practice due to its availability and ease of interpretation. Furthermore, the PaO_2_/FiO_2_ value is a determinant to guide the implementation of rescue therapies such as high positive end expiratory pressure (PEEP), neuromuscular blocking agents,^(^5^)^ prone positioning^(^6^)^ or extracorporeal membrane oxygenation.^(^7^)^ However, the PEEP level, the stabilization time after adjusting ventilatory settings, the time after ARDS onset and the FiO_2_ selected when obtaining arterial blood gases have all been shown to significantly influence the PaO_2_/FiO_2_ value.^(^8^)^

In clinical practice at the bedside, FiO_2_ selection is based on pulse oximeter saturation (SpO_2_). The most relevant ARDS clinical trials published in the last two decades set FiO_2_ to obtain a SpO_2_ between 88 - 95%.^(^5^-^7^,^9^)^ However, some controversies exist regarding the benefits and harms of liberal *versus* conservative oxygenation approaches in patients with classical ARDS during controlled mechanical ventilation.^(^10^,^11^)^ In this setting, the surviving sepsis campaign recently recommended a SpO_2_ between 92% and 96% in ARDS caused by COVID-19.^(^2^)^ Considering the particular pathophysiological features of SARS-CoV-2, FiO_2_ selection may considerably impact the oxygenation indices and affect clinical decisions. Furthermore, high concentrations of oxygen might alter the respiratory system mechanics through reabsorption atelectasis formation and augment the stress applied over the lung, thus promoting ventilator-induced lung injury.

The aim of this study was to compare gas exchange indices behavior by using liberal *versus* conservative oxygenation targets in patients with moderate to severe ARDS secondary to COVID-19 under invasive mechanical ventilation. Second, we assessed the influence of high FiO_2_ on respiratory system mechanics to evaluate the impact of reabsorption atelectasis on lung stress.

## METHODS

We conducted a prospective physiological study in the ICU of *Sanatorio Anchorena San Martín*. The local Review Board approved the protocol (committee’s reference number: 16/2020), and all of the patients’ next of kin signed informed consent forms. This is a preliminary report of clinicaltrial.gov NCT number: NCT04486729.

We included all consecutive patients admitted to our ICU aged over 18 years old with a confirmed diagnosis of COVID-19 (positive polymerase chain reaction through nasopharyngeal swab) and moderate to severe ARDS according to the Berlin definition.^(^3^)^ Other inclusion criteria were invasive mechanical ventilation requirement for less than 72 hours before enrollment and the need of neuromuscular blocking agents by medical decision. Based on previous physiological studies with similar methodologies and designs, we planned to include a sample size of 15 patients.^(^12^)^ The exclusion criteria were hemodynamic instability despite fluid resuscitation and vasopressor support, previous diagnosis of chronic obstructive pulmonary disease, no drained pneumothorax, intracranial hypertension, pregnancy, thoracic chest wall abnormalities, bronchopleural fistula and contraindications to esophageal catheter insertion.

Baseline characteristics and laboratory analysis of all patients were retrieved from our electronic clinical records. We collected the variables age, sex, number of days under invasive mechanical ventilation, Simplified Acute Physiology Score II (SAPS II) at admission, ARDS severity and sequential organ failure assessment (SOFA) score the day of enrollment.

### Respiratory mechanics

We evaluated respiratory system mechanics using a specific device and software (Fluxmed, MBMed®, Buenos Aires, Argentina) connected to a personal computer. The flow (F) and volume (Vol) were measured with a flow sensor provided by the manufacturer that was correctly calibrated. We inserted an esophageal balloon (MBMed® VA-A-008, nonlatex) 7 cm in length filled with 0.5mL of air. The correct position in the lower third of the esophagus was confirmed by the presence of cardiac artifacts and the occlusion test as previously described elsewhere.^(^13^)^ We performed end inspiratory and end expiratory occlusions of at least two seconds, and we evaluated the following variables: plateau pressure (P_plat_), driving airway pressure (ΔP_aw_), inspiratory esophageal pressure (P_es_ insp), expiratory esophageal pressure (Pes exp), driving esophageal pressure (ΔP_es_), inspiratory transpulmonary pressure using the direct method (P_L-direct_ insp), expiratory transpulmonary pressure (P_L_ exp), driving transpulmonary pressure (ΔP_L_) and inspiratory transpulmonary pressure using the elastance-derived method (P_L-elas_ insp) using the formula:

**Table:** 

$P_{L-\mathrm{elas}}\;\mathrm{insp}=\mathrm{Plateau}\;\mathrm{pressure}\;x\;\left(\mathrm{lung}\;\mathrm{elas}\tan\mathrm{ce}/\mathrm{respiratory}\;\mathrm{system}\;\mathrm{elas}\tan\mathrm{ce}\right).$

The respiratory system elastance (Ers), chest wall elastance (Ecw) and lung elastance (E_L_) were calculated with the following formulas:

**Table:** 

$E_{\mathrm{rs}}=\Delta P_{\mathrm{aw}}/\Delta \mathrm{Vol}(\mathrm{Expired}\;\mathrm{volume}\;\mathrm{in}\;L\mathrm{);}$
$E_{\mathrm{cw}}=\Delta P_{\mathrm{es}}/\Delta \mathrm{Vol}(L\mathrm{);}$
$E_{L}=\Delta P_{L}/\Delta \mathrm{Vol}(L\mathrm{).}$

### Oxygenation indices

The PaO_2_/FiO_2_ index was calculated as PaO_2_ (mmHg)/FiO_2_.^(^14^)^ To calculate other oxygenation indices, we used the equation of partial pressure of alveolar oxygen (PAO_2_) = ((Pbarometric - PvH_2_O) x FiO_2_) - PCO_2_/RQ,^(^14^)^ where Patm is the barometric pressure expressed in mmHg (760), PvH_2_O is the partial pressure of water steam expressed in mmHg (47), PaCO_2_ is the partial pressure of arterial carbon dioxide and RQ is the respiratory quotient (0.8). Once PAO_2_ was obtained, we calculated the indices alveolar-arterial oxygen pressure gradient (PA-aO_2_) and the quotient arterial/alveolar pressure of oxygen (Pa/AO_2_).

The functional shunt fraction was calculated based on venous admixture determination, considering central venous oxygen saturation (ScVO_2_) as an acceptable surrogate for mixed venous oxygen saturation: Qs/Qt = (CcO_2_ - CaO_2_)/(CcO_2_/CvO_2_), where CaO_2_, CvO_2_, and CcO_2_ are the arterial, venous and capillary oxygen contents, respectively.^(^14^)^ When available, mixed venous blood was obtained from a Swan Ganz catheter.

### Procedure

All patients were deeply sedated with propofol and fentanyl and paralyzed with atracurium. The subjects were ventilated in semirecumbent position in volume control mode with a tidal volume 6mL/kg of predicted body weight, square flow waveform with 0.3 seconds of end inspiratory pause, respiratory rate between 15 - 35 breaths per minute, aiming to achieve a pH between 7.20 - 7.45. The PEEP value was 5cmH_2_O.

We randomly applied two different FiO_2_ strategies to each patient: one strategy to achieve a liberal (96%) SpO_2_ and one to obtain a conservative (88 - 92%) SpO_2_, both periods evaluated on the same day. For randomization, we used the software available on the randomization.com website, and we used closed opaque envelopes. Each phase lasted 10 minutes, based on the study carried out by Cakar et al., in which they showed that 5 minutes was enough time to achieve a stable PaO_2_ level.^(^15^)^ After the end of each period, we obtained arterial and mixed venous blood samples and monitored the respiratory system mechanics. We did not use a washout period between each phase because of aspects related to the viability and safety of the patients included. Considering the critical status of our sample and the wide variety of factors that could affect arterial oxygenation (including basic care such as mobilization, aspiration of secretions, positional changes), extending the time of measurements would have led to limiting these interventions for longer periods of time, affecting the standard of care in our unit and the patient´s clinical status.

### Statistical analysis

Data are expressed as the mean ± standard deviation (SD) and number (percentage), as appropriate. The Shapiro-Wilk test was used to test normality. One sample Student`s t-test was used to assess the statistical significance of the difference between the two conditions when the data were normally distributed; otherwise, the Wilcoxon test was used. The results with a two-tailed p ≤ 0.05 were considered statistically significant. The statistical analysis was performed with R 4.0.3 (R Foundation for Statistical Computing - www.rproject.org) and the ggplot2 package.

## RESULTS

We enrolled 15 patients. The mean age was 55.6 years old, and 73.3% of patients were men with SAPS II 32 and SOFA 6.2 at admission (Table 1). Three subjects were classified as severe ARDS, and twelve were classified as having moderate ARDS. The median (interquartile range) of days between intubation and enrollment was 1 (1 - 3). The liberal oxygenation phase could not be completed in one patient due to desaturation despite using FiO_2_ 1.

The mean ± SD FiO_2_ and SpO_2_ for liberal and conservative oxygenation targets were 0.80 ± 0.19 and 96% ± 1 and 0.40 ± 0.13 and 89% ± 3, respectively.

**Figura 1:**
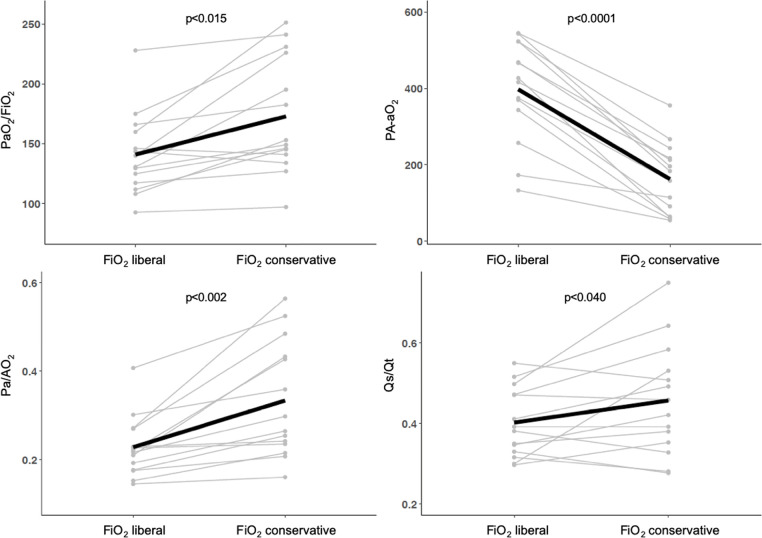
Oxygenation indices behavior with liberal and conservative oxygenation strategies.

The comparisons between oxygenation indices obtained with liberal versus conservative oxygenation targets are presented in figure 1. All of the indices were significantly affected by FiO_2_ selection. The PaO_2_/FiO_2_ deteriorated (FiO_2_ liberal; mean = 140.9 ± 34.0, FiO_2_ conservative; mean = 165 ± 54.4; p = 0.015), PA-aO_2_ increased (FiO_2_ liberal; mean = 397.5 ± 133; FiO_2_ conservative; mean = 190.4 ± 139.7; p < 0.001) and Pa/AO_2_ decreased (FiO_2_ liberal; mean = 0.22 ± 0.06; FiO_2_ conservative; mean = 0.31 ± 0.13; p = 0.002) significantly by using FiO_2_ to achieve SpO_2_ 96%. Conversely, the functional shunt fraction was reduced (FiO_2_ liberal; mean = 0.40 ± 0.08, FiO_2_ conservative; mean = 0.45 ± 0.13; p = 0.040).

**Table 1:** Baseline characteristics of the patients

Variables	
Demographic variables	
Female sex	4/15
Age	55.6 ± 9.4
APACHE II	13.1 ± 5
SAPS II	32 (10.8)
Respiratory variables	
Tidal volume (mL/kg)	6.1 ± 0.4
PEEP (cmH_2_O)	10.9 (10.5 - 12.5)
FiO_2_	0.45 (0.35 - 0.52)
Airway driving pressure (cmH_2_O)	10.5 (9.55 - 11.6)
Gas exchange	
PaO_2_/FiO_2_	147.4 (125.5 - 179)
Functional Qs/Qt	0.34 ± 0.11
Moderate ARDS, n/total	12/15
ICU mortality, (n/total)	6/15

APACHE II - Acute Physiology and Chronic Health Evaluation II; SAPS II - Simplified Acute Physiology Score; PEEP - positive end expiratory pressure; FIO_2_ - inspired fraction of oxygen; PaO_2_/FiO_2_ - partial pressure of oxygen/inspired fraction of oxygen; Qs/Qt - functional shunt fraction; ARDS - acute respiratory distress syndrome; ICU - intensive care unit. Data expressed as n/total, mean ± standard deviation, median (interquartile range).

The variables related to the respiratory mechanics are presented in table 2. There were no significant changes in mechanical variables between the conservative and liberal SpO_2_ strategies.

## DISCUSSION

Our findings show that adopting a liberal SpO_2_ target considerably affects the oxygenation indices, which may have implications not only in severity stratification of ARDS but also in the clinical decision-making process.

A proper stabilization time and standardized ventilatory settings have been shown to improve the severity stratification in classical ARDS.^(^6^)^ Villar et al. found that selecting an FiO_2_ of 0.5 with the aim of achieving an SpO_2_ not less than 88% allows to better identify patients at risk of death in comparison with higher fractions of inspired oxygen.^(^8^)^ In patients with a high percentage of shunt and low ventilation/perfusion (V/Q) units, increasing the oxygen supply significantly affects the gas exchange indices due to the marginal effect on PaO_2_ of higher concentrations of PAO_2_.^(^16^)^ Indeed, perfused and ventilated alveoli present limited capacity to increase CaO_2_, as explained by the classic behavior of the hemoglobin dissociation curve. Hence, deterioration in PA-aO_2_ and Pa/AO_2_ is somewhat expected considering that theoretical PAO_2_ will rise in the same proportion that FiO_2_ is changed (provided that PaCO_2_ is constant), but PO_2_ will not because deoxygenated blood leaving low V/Q units will mix with oxygenated blood coming from normal V/Q units.^(^17^)^ The patients included in our study presented a 40% functional shunt average, which explains why, even in COVID-19, where pathophysiological features may differ from classical ARDS, the application of high oxygen concentrations affected the gas exchange indices in a similar way to previous descriptions.^(^14^,^16^,^18^)^

PaO_2_/FiO_2_ - partial pressure of oxygen/inspired fraction of oxygen; PA-_a_O_2_ - alveolar-arterial oxygen pressure gradient; Pa/AO_2_ - quotient arterial/alveolar pressure of oxygen; Qs/Qt - functional shunt fraction.

The LOCO II trial recently found survival benefits at 90 days and fewer mesenteric ischemic events approaching a liberal oxygenation strategy in typical ARDS. The control group received lower PEEP and considerably less prone positioning trials, which could be explained by the fact that both interventions were decided based on the PaO_2_/FiO_2_ value.^(^11^)^ Our study suggests that using liberal SpO_2_ strategies may increase the need of rescue therapies to treat the refractory hypoxemia consequences of a remarkable deterioration of the PaO_2_/FiO_2_ index in this context. In our study, three patients changed their severity of ARDS from moderate to mild, and other three subjects increased their PaO_2_/FiO_2_ above 150mmHg only by using a lower FiO_2_, a situation that has been reported previously in non-COVID-19-related ARDS.^(^18^)^ Moreover, only two patients required less than 0.6 FiO_2_ to achieve at least an SpO_2_ of 96%, which should warns about the adverse effects of exposing the alveolar gas barrier to high concentrations of oxygen for long periods of time.

In conventional ARDS, atelectasis caused by superimposed pressure and lung volume reduction represent the main mechanisms of hypoxemia, showing a direct relationship between Qs/Qt and PaO_2_/FiO_2_ after adjusting for Crs.^(^19^)^ The same reasoning does not hold completely true for COVID-19-related ARDS. Our initial hypothesis was that high FiO_2_ would increase Qs/Qt secondary to reabsorption atelectasis and the reversal of hypoxic vasoconstriction.^(^20^)^ Our results showed the opposite, which could be explained by three potential reasons. First, respiratory system mechanics, in particular, lung stress (PL-elas insp and PL-direct insp) remained unchanged after increasing FiO_2_, which might indicate that atelectasis formation was not significant, possibly due to the limited time of exposure as well as the use of FiO_2_ lower than 100%. Second, the impairment of the normal mechanisms of hypoxemic vasoconstriction has been proposed as a possible cause to explain the profound hypoxemia in COVID-19 in the absence of significant alterations of respiratory mechanics;^(^21^-^23^)^ thus, increasing FiO_2_ could not have had considerable effects on vasomotor tone. Third, an adequate evaluation of shunt fraction implies the application of FiO_2_ 100%, a condition that was not accomplished because it was not the aim of our study. Setting FiO_2_ < 100% not only assesses the real shunt fraction but also includes those units with a low V/Q ratio in the Qs/Qt calculation.^(^17^)^ It is expected that increasing FiO_2_ will ameliorate the influence of low V/Q units, making the true shunt fraction more visible. Grasso et al. found a high proportion of Qs/Qt (> 40%) when assessing functional shunt with an FiO_2_ lower than 100%; when pure oxygen was used, the real shunt fraction was only 4%.^(^24^)^

**Table 2:** Respiratory mechanics behavior with liberal and conservative oxygenation strategies

Variable	FiO_2_ conservative (T1)	FiO_2_ liberal (T2)	T1 - T2 (95%CI)	p value
ΔP_aw_	10.0 ± 1.5	9.9 ± 1.5	-0.1 (-0.2 - 0.4)	0.579
P_plat_	16.1 ± 2.2	16.2 ± 2.3	-0.1 (-0.3 - 0.4)	0.682
ΔP_L_	8.1 ± 1.7	8.0 ± 1.8	0.1 (-0.4 - 0.7)	0.582
ΔP_es_	1.9 ± 0.9	1.9 ± 0.7	0.0 (-0.4 - 0.3)	0.258
P_L_ exp	-3.2 ± 3.4	-2.6 ± 3.2	-0.2 (-0.3 - 0.6)	0.768
P_L-elas_ insp	13.0 ± 2.6	13.1 ± 2.4	0.1 (-0.6 - 0.9)	0.741
P_L-direct_ insp	4.7 ± 3.1	5.3 ± 3.1	0.4 (-0.3 - 1.1)	0.280
E_rs_	26.2 ± 6.2	25.4 ± 5.1	0.2 (-0.7 - 1.1)	0.598
E_L_	20.8 ± 5.5	20.4 ± 5.0	-0.4 (-0.9 - 1.8)	0.498
Ecw	5.3 ± 2.7	4.9 ± 2.1	-0.2 (-1.1 - 0.7)	0.620
E_L_/Ers	0.79 ± 0.08	0.80 ± 0.08	0.01(-0.03 - 0.04)	0.839

FIO_2_ - inspired fraction of oxygen; 95%CI - 95% confidence interval; ΔP_aw_ - driving airway pressure; P_plat_ - plateau pressure; ΔP_L_ - driving transpulmonary pressure; ΔP_es_ - driving esophageal pressure; P_L_ exp - expiratory transpulmonary pressure; P_L-elas_ insp - inspiratory transpulmonary pressure using elastance derived method; P_L-direct_ insp - inspiratory transpulmonary pressure, direct method; E_rs_ -respiratory system elastance; E_L_ - lung elastance; Ecw - chest wall elastance. Data expressed in mean ± standard deviation and absolute difference (confidence interval).

Our study presents several limitations that must be addressed. First, the small number of patients enrolled in our study does not allow us to make conclusions regarding the best clinical strategy in terms of outcome benefits. On the other hand, cardiac output was not monitored during the protocol, and a reduction in the functional shunt when FiO_2_ was increased might be a feasible consequence of the reduction in cardiac output secondary to improvement in CaO_2_. Finally, all of the measurements were carried out with a PEEP of 5cmH_2_O and the behavior of gas exchange indices when varying FiO_2_ may be different with higher PEEP levels. However, this scenario is more physiologically attractive for assessing the effects of different FiO_2_ values considering that low PEEP exacerbates the loss of lung volume and increases the proportion of low V/Q units and functional shunt and, thus, the possible activation of hypoxia-induced vasoconstriction.^(^25^)^ In addition, the Berlin definition of ARDS not only defines but also stratifies the severity of the condition using a level of PEEP equal to or greater than 5cmH2O.^(^3^)^ In addition, several physiological studies have advocated using low PEEP levels to more accurately assess ARDS severity.^(^25^-^28^)^ Higher levels of PEEP might mask the severity of the underlying lung injury and impact the assessment of lung recruitability, and, therefore, hinder the prediction of the response to therapeutic interventions such as recruitment maneuvers or prone positioning.^(^25^)^

## CONCLUSION

A strategy aimed at liberal oxygenation targets significantly deteriorated gas exchange indices, except for functional shunt, compared with a conservative strategy in COVID-19 related acute respiratory distress syndrome during invasive mechanical ventilation. The respiratory system mechanics were not altered by the fraction of inspired oxygen strategy.
